# Movie Recommender Systems: Concepts, Methods, Challenges, and Future Directions

**DOI:** 10.3390/s22134904

**Published:** 2022-06-29

**Authors:** Sambandam Jayalakshmi, Narayanan Ganesh, Robert Čep, Janakiraman Senthil Murugan

**Affiliations:** 1 Department of Computer Science and Engineering, Vel Tech Multi Tech Dr. Rangarajan Dr. Sakunthala Engineering College, Chennai 600 062, India; jayalakshmi@veltechmultitech.org (S.J.); ganeshn@veltechmultitech.org (N.G.); 2 Department of Machining, Assembly and Engineering Metrology, Faculty of Mechanical Engineering, VSB-Technical University of Ostrava, 708 00 Ostrava, Czech Republic; 3 Department of Computer Science and Engineering, Vel Tech High Tech Dr. Rangarajan Dr. Sakunthala Engineering College, Chennai 600 062, India; j.senthilmurugan@velhightech.com

**Keywords:** movie recommender, filtering techniques, performance metrics, *K*-means, metaheuristics

## Abstract

Movie recommender systems are meant to give suggestions to the users based on the features they love the most. A highly performing movie recommendation will suggest movies that match the similarities with the highest degree of performance. This study conducts a systematic literature review on movie recommender systems. It highlights the filtering criteria in the recommender systems, algorithms implemented in movie recommender systems, the performance measurement criteria, the challenges in implementation, and recommendations for future research. Some of the most popular machine learning algorithms used in movie recommender systems such as *K*-means clustering, principal component analysis, and self-organizing maps with principal component analysis are discussed in detail. Special emphasis is given to research works performed using metaheuristic-based recommendation systems. The research aims to bring to light the advances made in developing the movie recommender systems, and what needs to be performed to reduce the current challenges in implementing the feasible solutions. The article will be helpful to researchers in the broad area of recommender systems as well as practicing data scientists involved in the implementation of such systems.

## 1. Introduction

Modern technology has revolutionized the volume, variety, and velocity at which data are generated. Digitalization of day-to-day experiences has led to the big data era. However, the enormous data have also led to the problem of information overload. Information overload may be defined as the state of being overwhelmed by the sheer volume of data presented to an average human for processing and decision making. Data mining methods can aid in obtaining and processing the relevant data and deal with the issue of information overload. Perhaps the most widely exploited tool among data mining methods is recommender systems.

Recommender systems work by assessing the available information about the likely patterns of the users and making suggestions from the information available [1]. The suggestions from the recommender systems help the system users find what is most suitable for them. Recommender systems are designed to ease product or service searches based on the least information available about the features [2]. A combination of various factors is used to assess the correlations in patterns and user characteristics to determine the best product suggestions for the customers [3].

The development of recommender systems depends on the field of application. The major application is in e-commerce websites where they suggest to the users the products or services based on the information available such as past search, age, gender, and other preferences [4]. They are also applied in job search platforms where the website suggests to a candidate the best possible positions fit for the skills. Since various industries have moved from an age of little available data to the era of big data, the junk information available is so much that it can delay the decision-making process. The recommender systems are typically made to ease the information search over the online systems so that the users find a more convenient way to connect to their preferences [5].

One of the applications of recommender systems is suggesting movies to watch to customers based on their preferences data. Movie recommender systems work by assessing the characteristic features of the users to make endorsements to the customers on what is best suited for them. It works by assessing the age, the previous preferences, gender, the content, context, and other demographic data to propose the movies. It checks the similarity among the users and items in the system to determine what could best fit the new user [6]. For example, a child will most likely receive recommendations for movies that children watch such as cartoons and animations based on the best similarity index for the children. Apart from that, children of various ages have different types of cartoons/animations to watch, and the recommender systems will propose the best depending on what other children of the same age are watching.

Movie recommender systems have helped the users overcome the chunk of information online to find only what is suited for them [7]. They use data mining techniques that match the similarities and help users find what is best suited for them [8]. Various criteria determine how the recommender systems work. The criteria are based on machine learning or deep learning algorithms that are used in matching the similarities before the suggestions are made. The algorithms achieve different levels of accuracy and require different computational times to retrieve the suggestions. Various computational algorithms have been proposed and used to increase the efficiency of recommender systems e [9]. However, each algorithm has its advantages and disadvantages; these make using the systems meet various needs based on their strengths. To reduce the limitations of each, the algorithms may be combined so that they perform better in making the recommendations [10].

This review paper aims to assess the challenges of recommender systems and make propositions to increase the accuracy of the systems. It assesses the recommendation approaches, the evaluation criteria of their efficiency, the challenges of these approaches, and possible solutions. A systematic literature review is conducted to determine the findings of the operational characteristics of the various recommendation approaches used and the performance criteria. The author aims to suggest the best solutions to make the approaches work better to achieve the operational expectations of the users.

The rest of the paper is arranged as follows: Section 2 details the methodology followed in this article. Section 3 describes different types of recommendation systems. Section 4 highlights some of the most popular machine learning algorithms used in movie recommender systems. Section 5 details the commonly used metaheuristic algorithms in movie recommendation tasks. Model metrics used for verifying the accuracy of recommendation systems are discussed in Section 6. Some common problems with recommendation systems are discussed in detail in Section 7. A critical discussion is presented in Section 8. Section 9 presents the concluding remarks and the limitations of the study.

## 2. Review Methodology

This section describes the method used in obtaining information for the literature review. Peer-reviewed sources were used to gather information about movie recommender systems. The databases used were EBSCO Academic Search Premier, ScienceDirect, IEEE Library, ResearchGate, SpringerLink, and the ACM Portal. Google Scholar was also used to find leads to specific aspects of recommender systems for review.

Search Descriptors: Some of the keywords used in finding information about the movie recommender systems were “movie recommender systems”, “movie personalization”, “algorithms used in movie recommender systems”, “filtering techniques in movie recommender systems”, and “machine learning model metrics and measurement criteria”.

**Figure 1:**
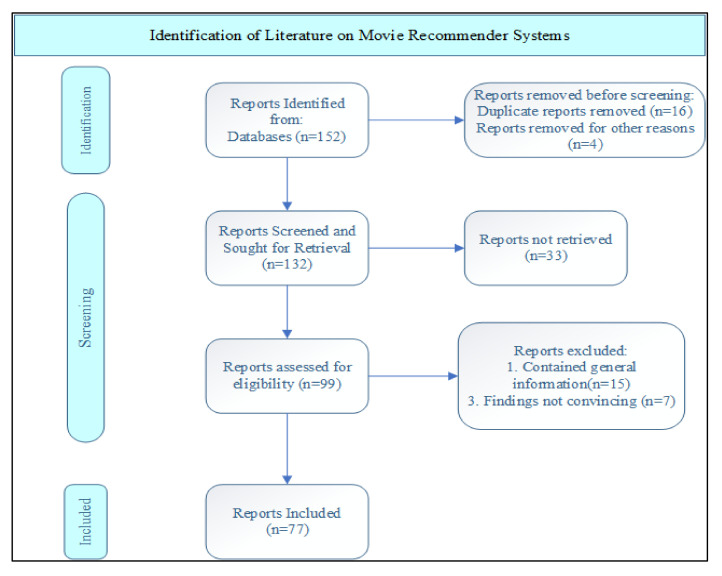
Steps in conducting the systematic review.

Inclusion Criteria: The inclusion criteria were papers that had information about recommender systems, the information had to be from published peer-reviewed sources. The paper abstracts were read to verify the validity of their information for use in this study. The exclusion criteria were papers that had grey literature on recommendation systems. The inclusion criteria for the articles and the methodology steps are summarized in Table 1 and Figure 1.

## 3. Movie Recommendation Systems

Movie recommendation work by filtering out data that is irrelevant and including only that which have matching characteristics or features [11]. As highlighted earlier, the world has moved from an era of scarcity of data online to an exponential growth in data. The systems work by manipulating the data to make sure it is efficient to drive data-driven decisions. In the jungle of available information about products, the systems need to evaluate what fits a certain customer and what does not. The systems go further in target and retargeting marketing to increase product viewership and hence increase the chance of the customers purchasing [12].

It is important for the developers to come up with systems that have higher performance characteristics and efficiency in matching the similarities in customer wants to seal the product sales or movie viewership [7]. The major types of filtering methods are collaborative filtering, content-based filtering, context-based filtering, and hybrid filtering.

### 3.1. Collaborative Filtering

Collaborative filtering works by matching the similarities in items and users. It looks at the characteristics of the users and the characteristics of the items the users have watched or searched for before [13]. In general, latent features obtained from rating matrices are looked at. In movie recommender systems, the recommendations are made based on the user information and what other people with similar user information are watching. For example, collaborative filtering in movie recommender systems picks the user demographic characteristics such as age, gender, and ethnicity [14]. Through these features, movie recommendations are made that match other people with similar demographic characteristics and previous user search history. Collaborative filtering suffers from a cold start if the user has not input any information, or the information is too little for any accurate clustering. In these cases, it does not know what to suggest [15]. The accuracy of the suggestion is also limited because people with similar demographic characteristics may not have similar preferences [16].

### 3.2. Content-Based Filtering

In contrast to collaborative filtering, content-based techniques employ user and item feature vectors to make recommendations. The fundamental differences between the two approaches are that content-based systems recommend items based on content features (no need for data about other users; recommendations about niche items, etc.) whereas collaborative filtering is based on user behaviour only and recommends items based on users with similar patterns (no domain knowledge; serendipity, etc.). A content-based filtering method works by making movie proposals to the user based on the content in the movies. It recognizes that clustering in the collaborative filtering recommendations may not match the preferences of the users [7]. The tastes and preferences of people with similar demographic characteristics are very different; what person X likes may not be similar to what person Y likes to watch. To solve this problem, content-based filtering algorithms give recommendations based on the contents of the movies [17]. In movie recommendations, some of the contents are the key characters and the genre of the movie.

### 3.3. Context-Based Filtering

This filtering technology is an improvement of the collaborative filtering method. It assumes that if person A and person B hold the same opinion on issue X, it is most likely that the same people will hold the same opinion/preference/thinking on a different issue Z. For example, if both people are attracted to Christmas movies from Netflix, it is most likely that they will still like Christmas movies by Showmax. The context-based filtering method recommends items with similar features or characteristics because the applications have just been extended to a different context [12]. It makes the same suggestions though the contexts are different. In most cases, web browsers import bookmarks and other settings when one upgrades from one browser to the next. This represents a change in context, since most of the settings and other items are imported into the new context, and the data available are used in making useful suggestions. Similarly, movie recommender systems may make a similar recommendation based on data from the previous context [18]. It is worth mentioning here about context-aware recommender systems (CARS), where the concept of context is well defined [19,20,21,22]. CARS acclimatize to the exact condition in which the recommended item will be used [23,24,25,26]. In this respect, CARS could avoid recommending a very long film to a user after a stressful day at work or suggest a romantic film if he/she is in the company of his/her partner.

### 3.4. Hybrid Filtering

This is a filtering technique that applies the concepts of all the other algorithms. It combines both collaborative filtering, content-based filtering, and context-based filtering to overcome the challenges of each method [10]. It is superior because it achieves higher performance in making the suggestions and also a faster computational time [11]. For instance, collaborative filtering may lack information about domain dependencies while content-based filtering lacks information about the preferences of the people [6,9,27]. A combination of these overcomes these challenges since user behaviour data and the content data are used to come up with recommendations.

## 4. Machine Leaning Algorithms for Movie Recommendation Systems

These are the algorithms that are used in filtering information and data mining so that the desired outcomes can be achieved. It is essential to understand the working of the information filtering methods so that the right algorithm is selected for the specific task in recommender systems [28].

### 4.1. K-Means Clustering

This is one of the simplest collaborative filtering approaches that categorizes the users based on their interests [29]. It is common for someone who wants to purchase an item to ask someone who has already purchased the product for their opinion. There is a higher chance that the influence of the current owner will affect the preferences and the tastes of the potentially new owner. Similarly, the algorithm compares the interesting features that can be associated with individuals that are classified to be within a group [30].

*K*-means clustering uses interests that are common among the users such as age, gender, movie time, history of the previous movies watched, etc. *K*-means clustering aims to group the features into clusters that represent the characteristics of the group [31]. If the classification is based on age, the probable *K*-means clustering will use children, teens, youth, and adult clustering methods. If a client falls within any of these age groups, movies are recommended based on what other people within that age group do. If the clustering depends on age, the closer an age is to the centroid age, the better the classification recommendation. The steps in the classification are measuring the similarity between the user and item features, selection of the neighbours, computing the prediction, and suggesting it [14].

#### 4.1.1. Measurement of the Similarities

The first step is finding the similarity in the user features that the new user has with the previous system users. The algorithm always has the basic classifications for a beginning, where the user can give inputs and the predictions can be made [32]. Common features in finding the similarities are age, previous history, and geographical locations. Other recommender systems in movie theatres, including the price, the time to watch the movies, etc., are used in coming up with the means (centroids) for clustering. The distance from the centroids can be based on a Pearson correlation, cosine-based similarities, or an adjustment of the cosine-based similarity. The calculation of the similarity may be item-based or user-based. Item-based computation finds the similarities based on the features in the movies that similar people liked. If it is user-based, the calculation of the centroids is based on the demographic features of the user [15].

The computation of the similarities between items or users is shown in the mathematical equations below:

(1) $$sim_{i,j}=\frac{\sum_{m\in \left(i\cap i\right)}\left(r_{i,m}-\bar{r_{i}}\right)\left(r_{j,m}-\bar{r_{j}}\right)}{\sqrt{\sum_{m\in \left(i\cap i\right)}{\left(r_{i,m}-\bar{r_{i}}\right)}^{2}}\times \sqrt{\sum_{m\in \left(i\cap i\right)}{\left(r_{j,m}-\bar{r_{j}}\right)}^{2}}}\;$$

The equation above computes the correlation between the user and the item; it computes the closeness of the value to the centroid value. It is assumed that the two items i∩j are the correlated features (items or users); the value $\bar{r_{j}}$ is the centroid feature, while the value $r_{i}$ is the value of the new user or new feature to be compared through correlation [33].

#### 4.1.2. Selection of the Neighbours

There is always a consideration when developing the algorithm. The key metrics are the accuracy to obtain and the running time of the algorithm. To increase the accuracy of an algorithm, a large number of neighbours, which increases the computational time of the algorithm, is required. If a smaller computational time is needed, accuracy will be compromised [34]. To strike a balance, the selection may be threshold-based or use the top-N technique. The threshold technique will run only a specific number (sample number that meets the threshold value) of assessments of the neighbours and predict if that threshold is reached. For example, if the population is 1000, the system will run a prediction from 100 samples and predict out of the 100 samples [35]. In the top-N technique, only the top number of similarities (N) is run rather than the whole population of neighbours. For example, it will select only the top 10 for suggestions based on the nearest neighbours rather than assessing the whole population [36].

#### 4.1.3. Prediction Computation

The computation of the subsequent predictions is based on the closest neighbours found in the system database. The prediction is obtained by the formula below:

(2) $$prediction_{u,\;i}=\frac{\sum_{n\in Neighbors}\left(r_{n,i}-\bar{r_{n}}\right)\;sim_{u,n}}{\sum_{n\in Neighbors}\;\;\left|\;sim_{u,n}\;\right|}+\bar{r_{u}}$$

The prediction or the nearest neighbour to the centroid (*K*-mean) is made. In the equation above, the *K*-means is represented by $\bar{r_{u}}$ while the correlation of the other variable on the right-hand side of the equation gives the nearest neighbour, both used in making the suggestion prediction [27].

#### 4.1.4. Limitations of *K*-Means Clustering

- *Cold-Start Problem*: This is a prediction problem that happens with a new user to the system. There is very little information about the user; hence it is difficult for the system to make any predictions until the user starts feeding some information that can be correlated to and suggestions made based on the user or previous item characteristics. The negative impact is the system accuracy is greatly reduced [6]. It is because of this problem that new and excellent movies are not recommended to users, or new users do not find what is best for them.
- *Sparsity in the dataset*: The recommendation system involves assessing a large amount of data in the movie database. The users only look for a few items in the database; they are not able to use and assess a significant portion of the database to effectively evaluate the features. Apart from that, the users do not rate the movies they watched in the system. It becomes hard for the system to determine if the user liked the movie they watched, or they never liked it because they never left any rating. The negative impact is leaving some of the best movies not recommended in the large dataset since they have not been rated by the user. Moreover, the threshold/top-N techniques leave out the best matching suggestions [37].
- *Scalability*: One of the challenges cited in the selection of the neighbours was balancing the computational time and the accuracy of the system. The *K*-means filtering technique is accurate when the database has a small number of movies to recommend or few users [38]. However, with an increase in the number of users and the number of movies, the computational time or the threshold number of items increases; therefore, the computational time increases [39]. To overcome this disadvantage, computation and training of the algorithm are performed offline so that when the systems are back online, recommendations are made easily [40].

The *K*-means filtering algorithm is the most basic collaborative filtering technique. It is from this technique that other filtering concepts are developed. The computation technique to arrive at the predictions may not be the same; the mode of working mimics the *K*-means nearest neighbour [41]. The other algorithms are developed to overcome the *K*-means clustering limitations.

### 4.2. Principal Component Analysis K-Means

This is a content-based movie filtering technique that improves on the *K*-means clustering technique. The major components in the movie are used to classify the movies before recommendations can be made to the customers. The *K*-means algorithm calculates the closeness of a feature to the centroid using the distance from the mean point. However, the principal component analysis creates a covariance matrix to calculate the eigenvectors and eigenvalues [42]. Therefore, it widens the scalability to find better comparisons to make the movie suggestions [43]. To illustrate this, assume a *K*-means algorithm computes the similarity of a single feature at a time. This implies the computational time and accuracy are compromised. However, using PCA, a covariance matrix of various features is created; hence the scalability is increased and computed faster. If there are similarities that fall within the matrix, they can be found easily, and its eigenvector is computed. Suggestions close to such an eigenvector are then made to recommend the movies [44].

#### Steps in Conducting Principal Component Analysis

- *Data formulation:* This is the first step where data is formulated and structured into tuples of dimension m × n. The possible tuples may depend on the user characteristics, the movie feature characteristics, or the combination of the user and feature characteristics [42]. The structure of the tuples is as given below in Table 2.

Where $r_{i}$ is the movie rating, $u_{i}$ is the user characteristics, and $i_{i}$ is the item characteristics (movie characteristics). A and B will give a 2D matrix of dimension *m × n* matrix, while C will give a 3D matrix of dimension m×n×o.

- *Calculation of the covariance matrix:* A covariance matrix of the dimension of the data formulated in the previous step is computed.
- *Calculation of the eigenvectors and eigenvalues*: The covariance matrix calculated will be a square matrix of the dimension of the data. It is used to compute the eigenvalues and eigenvectors which characterize the data. The computed eigenvectors are sorted in decreasing order according to the eigenvalues; a future vector is constructed [45].

### 4.3. Principal Component Analysis Self-Organizing Maps (PCA-SOM)

Self-organizing maps (SOMs) is a technique based on neural networks; it is an unsupervised learning technique, and there is no need for intervention of humans during the learning phase. It is vital in clustering data without knowing the class memberships in the input data [46]. The self-organizing feature map (SOFM) is known for detecting the features inherent in particular items which is important for the features in the movie recommender systems. SOM also uses topology-preserving mapping, which implies that the algorithm preserves the relative distance between all the points in the initial dataset [47]. Therefore, it effectively achieves the objective of transforming the arbitrary dimensions into a 1D or 2D discrete map. PCA is integrated with SOM because it is easier for the PCA to convert the matrices generated by SOM to eigenvectors and eigenvalues for ranking in the order of significance [48]. The steps in working out PCA-SOM are listed below:

1. Obtain data without any rankings or classifications;
2. Data modelling;
3. SOM classified the data using unsupervised learning to bring together that which has similarities in features;
4. PCA takes over from the classification achieved by SOM, checks the principal components, and comes up with further classifications of the dataset;
5. The decision to make the suggestion.

Initialization: Once the data are obtained, random values are chosen for the weight of the initial vectors. The weights of the vectors represent the neurons in the data, and their values are also computed [49].

Sampling: A known sample x is drawn from the input space with a known probability. This is the activation pattern that is applied to the lattice. This pattern maps the *x* dimension to be proportional to the m-pattern in the new lattice [49].

Similarity Matching: The best matching is found at time step-n using the minimum Euclidean distance between the neuron centroid.

(3) $$i\left(x\right)=\arg(\min\left|\left|x-w_{j}\right|\right|\;\mathrm{where}\;j=1,\;2,\ldots i$$

Updating: The synaptic weight of the neurons is adjusted using the formula below:

(4) $$w_{j}\;\left(n+1\right)=w_{j}\;\left(n\right)+\eta \left(n\right)h_{ji\left(x\right)}\;\left(n\right)(x\left(n\right)-w_{j}\left(n\right))$$

where $\eta \left(n\right)$ is the learning rate, $h_{ji\left(x\right)}\left(n\right)$ is the neighbourhood function of $i\left(x\right)$ winner neuron. These two are dynamic to obtain the optimum results.

Apply PCA: After the synaptic weights are derived from the minimum Euclidian distance from the formula above, the PCA process in creating the eigenvalues and eigenvectors is used further in processing the data to obtain a more accurate estimation [50].

Decision: After similarities are matched, the suggestions are made.

The great features of SOM that make it a good tool in recommender systems are:

Insights into the input space: The method uses unsupervised learning to classify the data by weight vectors and give output in a feature map. The cold starting is significantly reduced [51]. The user can then input data in light of the initial output features shown.

Topological arrangement: The feature map of SOM works by mapping the field of the input pattern to a spatial location in the output grid [52].

Density Matching: Once the input is fed into the system, any alterations in input distribution are equally represented in the output grid so that there will be a good representation of the highest density areas with the most matches and lower density areas with fewer matches [49].

Feature Selection: The SOM algorithm selects the best attributes for the non-linear distribution in the input data so that it can effectively match the similarities to the grids [50].

#### 4.3.1. Advantages

Since it is based on unsupervised learning, it automatically updates the features and functions [53]. It is flexible to new input because it learns by itself. It is suitable for unidentified new inputs, for example, new movies that have no ratings or new users where there is no data about them. The new movies may be recommended when the system extracts their features, and the new users will not experience a cold start because they have somewhere to begin on the output feature map [54]. It is also faster in computation since it easily organizes complex data and makes a good representation of the mapping for easy interpretation.

#### 4.3.2. Disadvantages

The major drawback is that feature classification may not be according to the expected output; therefore, the unsupervised learning classification algorithms have to be initialized often to maintain the relevance of the clustering [55].

## 5. Metaheuristic Algorithms for Movie Recommendation Systems

Metaheuristic algorithms are high-level methods or heuristics which have been developed to search, create, or select a heuristic that may produce a satisfactory solution for optimization problems. Metaheuristics find wide usage in almost all aspects of optimization problems. For example, metaheuristics have been used in design optimization [56,57,58,59], process optimization [60,61,62,63], structural optimization [64,65], knapsack problems [66,67], workflow scheduling [68], image segmentation [69,70,71], etc.

### 5.1. Genetic Algorithm

This is a hybrid filtering algorithm that uses the improved *K*-means clustering and is combined with the genetic algorithm (GA). It uses the PCA technique to partition the high dimensional space into clusters hence reducing the complexity of computations when making intelligent recommendations. The method has higher performance characteristics; hence it makes better recommendations. The steps of the recommendation system are outlined below:

#### 5.1.1. Data Preprocessing Using PCA

The first step is processing the data, extracting it from the original high dimensional space into a linear relatively low space with denser features that carry the information. The PCA feature extraction technique has been very effective. It combines the data represented by the principal component with the highest eigenvalue with the significant information after ranking them. The components with lower significance are ignored but components with higher significance are given prominence. After the linear reduction, only a selected number of components from the rank is fed to the GA-KM algorithm for classification.

#### 5.1.2. Enhanced *K*-Means Clustering Optimization by Generic Algorithms (GA-KM)

The objective is to make sure that the users/neighbours with like-minded interests or features are grouped. Therefore, it performs it in two stages which are *K*-means clustering and GA algorithms.

#### 5.1.3. *K*-Means Clustering

The technique, as discussed, centres its clusters around centroids based on the linear distance from the central feature. The correlation of distance from the central point determines the similarity index. If it is too similar, there is convergence; if there is a high dissimilarity, then the dataset is sparse. As discussed, it suffers a cold start, and its first centroid may be based on the local optimum rather than the global optimum. The steps in *K*-means clustering are selecting the centroids, assigning objects to the closest clusters, computing the sum of squared distances from the members in the cluster, and checking for convergence in the computed objects. The procedure for computation is similar to that discussed.

#### 5.1.4. Genetic Algorithm

This mimics biological evolution as explained by Darwin’s theory of evolution. The algorithm uses the population of individuals as chromosomes; the chromosomes represent possible solutions to the evolution problem [29]. Each of the chromosomes contains the genes with the survival ability. Therefore, through natural selection, the chromosomes with the highest quality genes have the highest chance of survival and are fit for reproduction for the next generation. The iterations are based on selection, crossover, and mutation. Selection picks just a proportion of the genes to breed for the next generation. Crossover swaps two parent chromosomes to be recombined into the offspring. Mutation randomly alters the value of a gene to produce offspring. The processes extend the diversity of the offspring. The processes end when the fitness conditions in the environment/context are met.

The GA algorithm is used to prevent premature convergence in the *K*-means algorithms. The centroids in *K*-means are considered the chromosomes; the fitness function to evaluate the quality of the solution is:

(5) $$f\;\left(chromosome\right)=\;\sum_{x_{i}\;\in \;X}min_{1\;\leq i\;\leq k}\;\left(dist\;\left(\;C_{i},\;x_{j}\right)\right)\;$$

The fitness value is the sum of the distances of the inner points to the cluster centres. The values are minimized to find the optimal partitions. To find the optimal partitions, the three generic operators precede the construction of the offspring based on the survival fitness principles; convergence occurs when the fitness criterion is satisfied [72]. The pseudocode of the algorithm is summarized below:

- Initialization

Parameter initialization: Set the maximum iterations, population size, cluster numbers, probability crossover, probability mutation, and fitness function to minimize the total distance of every sample to its nearest centre;

Population initialization: Randomly generate the initial population for each of the *k*-centers.

- Iterations

Selection operation;

Cross-over operation;

Mutation operations;

Obtain the initial *k*-centres with optimal fitness values;

*K*-means optimization: generate new clusters with *k*-centres.

When tested with the MovieLens dataset, the algorithm has better performance features especially in reducing the cold-start problem [29].

### 5.2. Firefly Algorithm

The algorithm is also bio-inspired from the fireflies and combines it with a fuzzy *C*-means clustering technique. In the natural world, the fireflies are pulled to the brightest firefly using the light signal. Each firefly pulls the other, but the brightest has the highest attractiveness, and other fireflies are clustered around it. Similarly, the algorithm centres its suggestion on features of the users with the highest attractiveness (highest user ratings) [55]. If a movie has the highest rating from many users, the movie recommender system will make subsequent recommendations based on movies rated highest by users with similar characteristics [73]. The algorithm for the recommender system is highlighted below:

- All the fireflies are unisexual, and every firefly pulls to another firefly.
- The attractiveness of a firefly depends on its brightness, and the other fireflies will be pulled closer to the brighter one (feature reduction using the firefly algorithm).
- This brightness is related to a primary function in the FCM.
- FCM allocates memberships and utilizes them to show data elements from one cluster to another.
- The FCM separates a finite set of elements $\left(X=X_{1}\ldots .\;X_{n}\right)$ from the memberships into a set of *c* fuzzy clusters; hence it comes up with a list of cluster centres $C=\left\{C_{1}\ldots \;C_{2}\;\right\}$. The partition matrix $W=W_{i}\;\left[0,1\right];\;i=1\ldots \;n;\;j=1\ldots c$ expresses the degree to which each element $X_{i}$ is placed into a cluster $C_{j}$. The aim is to reduce the objective function to optimal.
  (6) $$C^{arg}\;\overset{C}{\underset{i=1}{\sum }}\overset{C}{\underset{j=1}{\sum }}W_{ij}^{M}\;{\left|X_{i}-C_{j}\right|}^{2}\;$$
- Then the fuzzy *C*-means clustering
  (7) $$f_{lm}^{m}=\frac{1}{\sum_{K=1}^{C}}\;{\left[\frac{\left|x_{i}-c_{m}\right|}{\left|x_{i}-c_{k}\;\right|}\right]}^{\frac{2}{n-1}}\;$$

The recommender system efficiency and performance are generally higher than the traditional *K*-means clustering. 

### 5.3. Artificial Bee Colony

This is a bioinspired algorithm that makes recommendations based on the workings of the bees in finding flowers for the best nectar [74]. The bees are mainly divided into two groups. Scouting bees go out to scout flowers with the best nectar, and the employee bees follow after the best flowers have been found [53]. It is worth noting that several scouting bees are sent out and come back with information to the hive regarding the quality of the nectar found. The employee bees will filter out the low-quality nectar from the information and follow the scout bee to the source of the best nectar. Similarly, the artificial bee colony in recommender systems works as an improvement of the *K*-means clustering algorithm [75]. In the *K*-means algorithm, an assumption is made that the data is based on a centroid where the closeness of the feature to the centroid feature determines the recommendation. In an artificial bee colony, there are many centroids (just as there are many flowers), and information from or to these centroids will bring a variety. From this variety, the user may choose what is best suited for them; henceforth, the recommendation system will bring recommendations close to the centroid chosen [76]. It is a good method to solve the sparsity, scalability and cold-starting problem. The user will choose the best suited feature from the first random set of options available. Subsequent recommendations will depend on the *K*-means around that particular choice. The steps are summarized below:

- Initialize the system users and movies in a matrix;
- Use the *K*-means clustering to find several centroids of various product features. This finds several centroids for clustering;
- Selection of the nearest clusters;
- Calculation of the estimated rating values from the user history;
- Use the artificial bee colony to select the closest to user likes based on ratings and features;
- Reclassification of the users for further iterations;
- Coming up with the recommendations.

ABC determines the community of vectors that explore the similarities in the neighbours. The objective function is then continually reduced when narrowing down to the nearest possibilities [77]. The aim is to minimize the objective function below:

(8) $$f\vec{\left(z\right)},\;\vec{\left(z\right)}=\left(\;z_{1},\;z_{2},\;\ldots ,\;z_{i},\;\ldots ,\;z_{n-1},z_{n}\right)\in R_{n}$$

The objective function is controlled by succeeding iterations determined by the detecting vector $\vec{\left(z\right)}$ as below:

(9) $$L_{i}\;\leq z_{i}\leq U_{i},\;i=1,\ldots ,n\;\mathrm{for}\;gy\vec{\left(z\right)}\;\leq 0\;\mathrm{for}\;y=1,\ldots ,\;p;\mathrm{and}\;hy\;\vec{\left(z\right)}=0\;\mathrm{for}\;y=p+1,\ldots ,\;q$$

From the succeeding iterations, the points with the most similar features are selected, and the system recommends the movies to the user [78]. For instance, an initial allocation of the centroids may be classified as horror movies, thrillers, comedy, or thrillers. If the client selects thriller movies, a further classification may be Hollywood thriller, Bollywood thriller, etc. If the client selects any from these, the subsequent recommendations will be based on this particular centroid [74]. As seen, it has an advantage because there are always initial centroids that the user can select that further narrow down the selection. Optimization is reduced by the detecting vector to optimize the suggestion to the most viable suggestions [79]. 

### 5.4. Cuckoo Search

The cuckoo search algorithm is a combination of *K*-means clustering and the use of Levy’s flight function. In this process, the *K*-means algorithm divides the MovieLens Dataset into different clusters. This is performed using randomly selected centroids [14]. Measures such as the Euclidian distance and cosines are used to find the distance between centroids, and the features and/or users are reassigned to the closest cluster with similar characteristics.

The cuckoo search algorithm gets its inspiration from the cuckoo bird. The cuckoo bird does not sit on eggs to hatch; rather, it searches for the best nest with optimal conditions and lays eggs for the host bird to sit on to hatch [80]. If the host bird identifies the egg, it may throw it away or abandon the whole nest. Similarly, in the recommender algorithm, if the centroid does not present the optimal solution, the centroid is abandoned for a new iteration until no re-assignment happens. The pseudocode for the recommender system is outlined in the procedure below:

#### 5.4.1. *K*-Means Clustering

- Initialize the number of *k* clusters;
- Random selection of centroids using *K*-means clustering;
- While no centroid is changed, assign each data point to the closest centroid and calculate the new centroids;
- Assign data points to the closest cluster mean.

#### 5.4.2. Cuckoo Search Algorithm

- Begin the fitness function $f\left(x\right),\;X_{i}=\left(\;x_{1},\;x_{2}\ldots \right)$;
- Initialize the random population of n host nests (centroids from *K*-means);
- Calculate the fitness function value for each nest;
- Find the ith cuckoo randomly by Levy flights, and calculate its fitness, *Fi*;
- Select a nest (centroid);
- If ($F_{j}>F_{i})$; replace j with the new solution;
- The unqualified nests (centroids) are abandoned and new ones built by Levy flights function;
- The best solutions found are ranked and suggested to the client.

The cuckoo search algorithm has higher precision, recall, and a lower *MAE* than the PCA-*K*-means, hence higher performance characteristics. The computations can also be performed offline so that recommendations are made when the system is online, making it faster to make suggestions to the user. 

### 5.5. Grey Wolf Optimizer

This is a recommendation system that is based on mimicking the leadership and hunting tactics of the grey wolfs [81]. The algorithm first conducts feature selection using the grey wolf optimizer (GWO) method, before clustering using the FCM method [82]. The algorithm pseudocode is listed below:

- Load the GWO culture;
- Initialize the coefficient points r, Q and R;
- The appropriateness of each explorer is estimated $X_{\alpha }\;X_{\beta }\;X_{\delta }$;
- Carry out iterations to determine the appropriateness of entire explorer negotiators;
- Return $X_{\alpha }$ representing the position of centroids by GWO;
- Randomly select the cluster centres based on fuzzy means;
- Load the fuzzy clustering formula matrix and estimate $f_{lm}^{m}$ as in the formula below:
  (10) $$f_{lm}^{m}=\frac{1}{\sum_{K=1}^{C}}\;{\left[\frac{\left|x_{i}-c_{m}\right|}{\left|x_{i}-c_{k}\;\right|}\right]}^{\frac{2}{n-1}}$$
- Determine midpoints B*(k)* = [*c_m_*] with F*(k)*; continue with iterations until ||F(*k* + 1) − F(*k*))) < ε;
- Return to the newly formed cluster centres and make recommendations based on the cluster centres.

This recommender system has a relatively better performance. 

### 5.6. Other Metaheuristic Algorithms

Some researchers have used other metaheuristic algorithms to develop movie recommender systems. For example, Papneja et al. [83] developed a movie recommendation using a whale optimization algorithm. Tripathi et al. [84] hybridized a map-reduce-based tournament along with a WOA to achieve a superior recommendation experience.

## 6. Model Metrics

Various aspects have to be measured apart from the accuracy to make sure that the algorism makes the right predictions. For example, the algorithms may be highly accurate but have too much logarithmic loss. Accuracy is not the only metric to determine the performance efficiency of a model. The metrics are discussed below:

### 6.1. Mean Absolute Error (MAE)

This is the average difference between the predicted values and the original values. In our case, it is the average difference between the choice of the movie by the customer (user) from the suggestion made (prediction). It gives the variation between the suggestion and what the customer chose. The only disadvantage is it does not give the direction of the error [85]. Generally, a low mean absolute error is desirable. The mathematical formula for *MAE* is shown below:

(11) $$MAE(y,\bar{y}=\frac{1}{n_{s}}\;\overset{n_{s\;}-1}{\underset{i=0}{\sum }}\left|y_{i}-{\bar{y}}_{i}\right|$$

where $n_{s}$ is the number of samples, ${\bar{y}}_{i}$ is the predicted suggestion, and $y_{i}$ is the true feature that the user picks/wants.

### 6.2. Mean Squared Error (MSE)

This gives the square of the *MAE* (the square of the average difference between the original values and the predicted values). The advantage is that it makes the large errors more pronounced so that the model focuses on the large errors and their causes [86]. In addition, it is easier to model the linear programming models in the computation of the slope using the mean absolute error since the differences will be clearer. The formula for the MSE is shown below:

(12) $$MAE(y,\bar{y}=\frac{1}{n_{s}}\;\overset{n_{s}-1}{\underset{i=0}{\sum }}{\left(y_{i}-{\bar{y}}_{i}\;\right)}^{2}$$

### 6.3. Log Loss

This is a cross-entropy loss given by probability estimates. It is used in neural networks and recommender system optimizations. It calculates the probability of the suggestions rather than giving only discrete predictions, especially during the ranking of the suggestions [35,87].

### 6.4. Confusion Matrix

This is one of the most used metrics in determining the accuracy of a model. It is mainly used for classification problems, especially when the outputs expected should have more classifications [34]. The various characteristics of the confusion matrix are shown in Table 3.

As noted earlier, the movies are clustered based on the features or the users. In clustering, the true represents the actual classification of the movie, while the predicted gives the predicted classification of the movie before recommendation [88]. For example, a movie may be classified as a comedy when it is a thriller movie. The user may choose to think it is a comedy because of the characters only to find it is a thriller movie. The movie may be classified as a thriller, and it is a thriller; hence the users get what they want. Such variations happen in the movie classification; hence there is a need for accurate predictions. 

***True Positive:*** This points to a case in the recommender system where the actual suggestion was positive, and the client selection of the movie was positive, i.e., the system suggests what the client needed. An example is when the movie is classified as a comedy when the client needed comedy and selects it [89].

***True Negative:*** This happens when the actual classification is negative, and the prediction is also negative. In our movie recommendation example, if the movie is not a comedy and our algorithm does not classify it as comedy, this output is termed as true negative [90].

***False Positive:*** This happens when the actual classification is negative, but the system predicts it as positive [91]. In the movie recommendation system example, the actual case may be the movie is not a comedy; yet the prediction algorithm classifies it as comedy. The actual movie is not a comedy, hence negative, but the prediction is comedy, hence the term positive.

***False Negative:*** This happens when the actual data is true (positive), but the prediction is negative (false) [87]. The actual classification is true; yet the system predicts it as negative. In the movie recommender systems, the actual movie is a comedy, but the system predicts not comedy. 

### 6.5. Precision

This assesses how many of the true positives are true positives. It gives a fraction of the true positive predictions to the total positive predictions [92]. The mathematical format is shown below:

(13) $$\mathrm{Precision}=\frac{\mathrm{TP}}{\mathrm{FP}\;+\;\mathrm{TP}}$$

### 6.6. Recall/Sensitivity

This is used as the fraction of true values from the total true values [35]. In our recommender systems, it gives a fraction of what is classified as comedy out of a total of what is comedy. The fraction of the true values out of the total true values is mathematically modelled as below. Note that false negative is true, but the algorithm classified it as else:

(14) $$\mathrm{Recall}=\frac{\mathrm{TP}}{\mathrm{TP}+\mathrm{FN}}$$

Precision focuses on capturing the classifications correctly, while recall focuses on whether the system is able to capture the features we want though they may not be correctly captured [34]. 

### 6.7. Accuracy

This is the fraction of correct predictions with the total predictions. The accuracy is mathematically modelled as below:

(15) $$\mathrm{accuracy}=\frac{1}{n_{s}}\;\;\overset{n_{s}-1}{\underset{i=0}{\sum }}1\left({\bar{y}}_{i}=y_{i}\right)\;\;\;$$

(16) $$\mathrm{accuracy}=\frac{\mathrm{correct}\;\mathrm{predictions}}{\mathrm{total}\;\mathrm{predictions}}=\frac{\mathrm{TP}\;+\;\mathrm{TN}}{\mathrm{TP}\;+\;\mathrm{TN}\;+\;\mathrm{FP}\;+\;\mathrm{FN}}$$

Accuracy as a metric should be used only when the data are balanced and have various classes [93]. It should not be used as a metric when the data are skewed (have a majority of only one class). For example, if the data are made of 100 movies, and only 5 movies are comedies, the rest are different genres such as thrillers. If the algorithm wrongly predicts all the movies as thrillers, it will be 95% accurate because 95% are thrillers, and only five are comedies. However, from a rational standpoint, the algorithm failed to classify comedy movies. In a recommendation system, the client would think that none of the movies in the datasets is comedy and not watch; yet there are five top-rated comedy movies. Therefore, accuracy should be used when the dataset is well-balanced.

### 6.8. F_1_

This is simply the harmonic mean of precision and recall. It shows how precise the system was and how it never missed significant instances. If the F_1_ score is high, the model performance is high [94]. The mathematical formula for precision is shown below:

(17) $$F_{1}=2\;\frac{\mathrm{precision}\;\times \;\mathrm{recall}}{\mathrm{precision}\;+\;\mathrm{recall}}$$

### 6.9. Computational Time

This is the time that the algorithm takes to come up with the final solution in the prediction. If the systems take long, they may be unreliable if the users want an immediate response before they select the movies to watch. The algorithms should ensure that the best results are found within the shorted period possible [53,95]. A high-performing system returns the most efficient results within a relatively short period. It increases its reliability and dependency. Sometimes the data to be analyzed may be too large to give immediate results. To overcome this limitation, the computations are performed when the system is offline so that the output is shown when it is back online for effective prediction suggestions [96].

## 7. Problems Associated with Movie Recommender Systems

### 7.1. Cold Start

The best target audience for a recommendation always depends on the previous user characteristics and the features of the products they watched. A comparison is always made on the characteristics of the user was and the features of the movie and the rating given to the movie. However, in some instances, there are no user characteristics that can be used for a recommendation if the user is new, and nothing is known about them [97]. Sometimes the user may not be new but has used a different device when accessing the movies’ websites hence there are no stored cookies that can trace the user history.

A cold-start problem occurs when the recommender system is not able to make any suggestions to the user because the user is new or there is no information available about the user [98]. The problem is common in collaborative filtering which uses only user details to make recommendations of the best movie. The problem is overcome by using content-based filtering, context-based filtering and hybrid filtering. In content-based filtering, the movies are classified by the features such as the main characters, the genre etc. The new user will select any genre based on the content. A context-based filter is based on some of the user information derived from the device such as location, and the operating system, and correlates them with what other users from similar contexts are using. In hybrid filtering, the content, context, and user characteristics are used, therefore, if the recommender system does not have any information about the user, it will use the content and context to make the first recommendations [99]. The subsequent recommendations will depend on the hints of information available.

### 7.2. Accuracy

If the database for the recommender systems has few movies, the system will have higher accuracy. If the database is large, there tends to be a lower accuracy because the pool of information searched is too large. To counter the problem, the *K*-means algorithm reduces the computational time by restricting the computation to a certain number of iterations or selecting only the top-N number of movies for recommendations [100]. However, if some of the movies have never been rated, they are likely to be biased in the searches [101].

To increase the system accuracy in making the recommendations, some of the algorithms have employed sophisticated search criteria that will conduct a thorough search and match the product features to user and item characteristics [102]. In addition, two or more algorithms are combined to allow the perfect user and feature analysis and come up with the desired output. Some of the classification processes such as the PCA-SOM conducts the logarithmic computations offline so that they give recommendations easily when they are online. It reduces the computational time and increases the recommender system accuracy. In modern recommender systems, cold-start problems and the accuracy is solved by having a dialog box where the users can type in the features they need, and recommendations will be given according to what matches the search words [103].

### 7.3. Diversity

New movies in the recommender systems rarely appear among those that are suggested to the users. The new excellent movies may end up not being watched because of the lack of being rated by the users. Some of the excellent movies also may not be rated, leaving the recommendation system blank about whether the movies are great for a specific class of watchers or not. To overcome some of these challenges for new movies and movies that are not rated by the users, the diversity aspect is introduced by the recommender systems. In diversification, the new movies or unrated movies are given priority so that they can be noticed by the users [104]. If they are pleasing, they will be rated and watched more. From this information, the recommender systems will make subsequent decisions on whether to recommend the movie or archive it. From the number of watches, it will also classify the movie according to the features or user characteristics [102]. Diversity is often used to recommend new debut movies to increase their marketability and presence. It increases the diversity of the user to try out new features or new products.

### 7.4. Scalability

While sparsity and diversity aim to increase the chances of movies with new features appearing in top searches, scalability aims to solve the problem of increased computational time and increase the performance of the recommender system. Scalability ensures that there is a balance obtained between accuracy and computational time. If it is necessary, some of the classification computations are performed beforehand so that by the time the user comes to select an item to watch, the system makes an almost immediate recommendation with high levels of efficiency [105].

### 7.5. Sparsity

Sparsity in the movie data relates to the large volume of movie data in the system, but the users only utilize a few of the features or resources. It is common in *K*-means clustering where the data is interpolated linearly and gives fewer perspectives to the non-linear data [106]. Recommendation systems may sometimes be biased by only suggesting the most rated or the most liked movies based on a limited assessment of all the possible cluster features. By using the top-N theory to make the recommendation, those that do not meet the threshold of these can find better algorithms that consider the sparsity of information available [107]. Some of the methods such as PCA-SOM map all the features on a lattice; hence the user can find most of the features. WOA also widens the scope of the search by using both linear and orthogonal systems to find the desirable features and make recommendations that are sparser and more diverse. Generally, implementing systems that consider non-linearly related data is efficient [108,109].

## 8. Discussions

The current movie recommendation systems have to work in contexts where there is so much data to be considered before making recommendations. Both user and context information are so varied that the accuracy and precision of the systems are brought to real tests. For example, most of the user information is shared through social media platforms to generate interest in the movies. The MovieLens dataset was created approximately 20 years ago when there was little or no developments in the use of social media where users share movie information to create interest. However, current technologies need to analyze the content, context. and user characteristics in social media platforms to recommend the right movies to the customers. Some companies have taken steps to integrate analytics in their recommender system algorithms. They ask the customer to connect to their social media accounts such as Twitter, YouTube, and Meta not only for advertising but also to analyze the activity of the user on these social media accounts to recommend the best movies for them. Through connecting to these platforms, they analyze the previous history of the user and recommend appropriate movies. This significantly reduces the cold-start problem since new user information can be obtained.

Context-based filtering is gaining traction in the movie recommender systems. It has been adequately used in product recommendations on e-commerce platforms., for example, the most discounted products during black Fridays, the holiday products during Christmas seasons, etc. Movie recommender systems that integrate time stamps to recommend the best movies in various contexts should be studied and developed. For example, it will help recommend movies for children learning during the day and children lullaby movies when it is time to sleep at night.

There are various advances in the use of blockchain technology, and some of these applications may affect the efficacy of algorithms in movie recommender systems. Blockchain technology enhances user privacy through user data encryption; yet collaborative filtering depends on the availability of user information so that it can match the features and characteristics before making recommendations. If user information is concealed by the blockchain systems, the algorithms have to use advanced methods to prevent a decline in the accuracies such as the use of context and content-based filtering.

## 9. Conclusions

In this article, movie recommender systems have been described and classified. The various types of recommender systems are introduced and discussed. Special emphasis is given to explain in detail the various machine learning and metaheuristic algorithms commonly deployed in movie recommendation research. The various model metrics that summarize the quality of the model are discussed at length. The problems associated with movie recommender systems are also summarized in a structured way and discussed. A total of 77 articles strictly on the area of movie recommender systems are included in the study, and their major conclusions are presented. In addition, 32 other related articles on metaheuristics and recommender systems (not for movies) are also introduced in various sections to present a coherent and meaningful review. One of the limitations of the study is that the Scopus and Web of Science databases were not directly used for selecting the articles for review. In contrast, EBSCO Academic Search Premier, ScienceDirect, IEEE Library, ResearchGate, SpringerLink and the ACM Portal were used for the literature search. Nevertheless, more than 80% of the reviewed papers were found to be indexed in Scopus while more than 60% were available in the Web of Science database.

## Figures and Tables

**Table 1:** Selection criteria for including sources in this review.

Item	Search Criteria	Number of Articles	Selected Articles
Filtering Methods	Collaborative filtering, Content-based filtering, context-based filtering, hybrid filtering	35	20
Movie Recommender System Algorithms	*K*-means clustering	21	12
	Principal Component Analysis	20	8
	PCA-Self Organizing Maps	18	10
	Genetic Algorithm	2	2
	Fireflies	2	2
	Artificial Bee Colony	13	7
	Cuckoo Search	8	5
	Grey Wolf Optimizer	2	2
Measurement metrics	Mean Absolute Error, Precision, Accuracy, Recall, Computational Time, F1, Log loss, Mean Squared Error	20	8
Recommender System Problems	Cold start, scalability, diversity, accuracy, sparsity	17	7

**Table 2:** Structure of the tuples.

Tuple	Tuple Structure
A	$r_{i}\;\times u_{i}$
B	$r_{i}\;\times i_{i}$
C	$r_{i}\;\times u_{i}\times i_{i}$

**Table 3:** Characteristics of a confusion matrix.

	Positive	Negative
Positive	True Positive (TP)	False Negative (FN)
Negative	False Positive (FP)	True Negative (TN)

## Data Availability

Not applicable.
